# 5G planar branch line coupler design based on the analysis of dielectric constant, loss tangent and quality factor at high frequency

**DOI:** 10.1038/s41598-020-72444-2

**Published:** 2020-09-30

**Authors:** Nor Azimah Mohd Shukor, Norhudah Seman

**Affiliations:** Wireless Communication Centre (WCC), School of Electrical Engineering, Universiti Teknologi Malaysia (UTM), 81310 UTM Johor Bahru, Johor Malaysia

**Keywords:** Electrical and electronic engineering, Materials science

## Abstract

This study focuses on the effect of different dielectric properties in the design of 3-dB planar branch line coupler (BLC) using RT5880, RO4350, TMM4 and RT6010, particularly at high frequency of 26 GHz, the fifth generation (5G) operating frequency. The analysis conducted in this study is based on the dielectric constant, loss tangent and quality factor (Q-factor) associated with the dielectric properties of the substrate materials. Accordingly, the substrate that displayed the best performance for high frequency application had the lowest dielectric constant, lowest loss tangent and highest Q-factor (i.e., RT5880), and it was chosen to enhance our proposed 3-dB BLC. This enhanced 3-dB BLC was designed with the inclusion of microstrip-slot stub impedance at each port for bandwidth enhancement, and the proposed prototype had dimensions of 29.9 mm × 19.9 mm. The design and analysis of the proposed 3-dB BLC were accomplished by employing CST Microwave Studio. The performance of scattering parameters and the phase difference of the proposed BLC were then assessed and verified through laboratory measurement.

## Introduction

Due to the rapid advancements in wireless communications, a sophisticated communication system that includes high-performance devices necessary to deploy next (i.e., fifth) generation (5G) technologies. This system requires devices that further perfect wireless communication between anything, anywhere and at any time^1^. The 5G were deployed in several countries including South Korea, the United Kingdom, Germany and the United States and many more countries are expected to deploy 5G soon and the high demand for 5G technology, which require 24/7 ubiquitously high-speed connectivity become a significant challenge for researchers and engineers involved in the development of radio frequency (RF) and microwave components, antenna design, and network planning. Butler matrix design is particularly high-demand in 5G application because the Butler matrix can be employed to support the control capability of beam-forming and electric scanning antennas^2^.

The Butler matrix was first introduced by Jesse Butler and Ralph Lowe^3^ and the beam-forming network creates multiple fixed overlapping beams to cover the designated angular area^4^. Since it is an N × N passive reciprocal network, it can be used to transmit and receive signals^5,6^. There are several properties associated with the Butler matrix, including inputs that are isolated from each other, phases of N outputs that are linear with respect to position and a phase increment between the outputs that depends on the selection of input^4^. The Butler matrix is also capable of beam-steering with uniform amplitude and adequate phase difference between consecutive output ports^5^. The power at the input port of the Butler matrix is equally distributed to the output ports^4^ and the configuration of the Butler matrix essentially consists of passive components such as a branch line coupler (BLC), a phase shifter and crossover^7,8^. A non-conventional configuration BM has been proposed by Babale et al.^9^, where the BM was constructed without using crossover and phase shifter, however it was not practical for cascaded BM due to the position of input and output ports, which may lead to the connection issues. As the most critical component in the Butler matrix configuration, the BLC designed during the first stage must display good performance. Since a BLC is only capable offering narrowband performance, many studies related to bandwidth improvement have been performed. The defect ground structure (DGS) technique can be employed under the branch arm of the BLC for to improve bandwidth^10^. Employing the DGS technique able to enhance the bandwidth of the BLC design^10^ by 8.2% compared to conventional BLCs.

Then, a dumbbell-shaped DGS was etched on the ground plane of another BLC^11^ design. In the design, a 120Ω transmission line was used to replace the quarter-wavelength 150Ω transmission line. The performance of this design indicated that the good results could be achieved within a frequency range of 1.85–3.35 GHz with a bandwidth enhancement of approximately 20%. However, the disadvantage of using a DGS is that it is only suitable to be implemented with the high line impedance. Another technique that use a slotted line on a BLC was introduced to further improved bandwidth^12^. The slotted line was placed at the ground plane and positioned beneath the vertical branches of the BLC design. It was concluded that slot dimensions (i.e., *W* × *L*) of 0.7 mm × 11.5 mm resulted in the best BLC performance within a frequency range of 3.5–4.7 GHz^12^. This BLC design^12^ has improved bandwidth by 80% compared to conventional BLCs. Another 3-dB BLC with improved bandwidth proposed by Jin and Xu^13^ by introducing the artificial magnetic conductor (AMC). The design comprised of a spacer with hollow interspace in the middle layer that sandwiched by AMC patches at the upper layer and microstrip coupler which printed on the upper surface of the bottom substrate layer. This multilayered coupler structure faced misalignment between each layer and air gap in the fabrication stage that led to a significant impact on the performance for high frequency applications. Thus, the implementation of a single substrate in the coupler design at high frequency is preferable.

Another crucial aspect in designing the coupler is the planar dielectric material, which is also known as the substrate. The parameters of the substrate are not only focused on the degree of design miniaturization but also influences the quality factor (Q-factor) of the components. The effect of different dielectric constants, *ɛ*_*r*_ towards coupler size has been presented by Letavin Denis et al.^14^ concerning Rogers RT (*ɛ*_*r*_ = 3) and FR-4 (*ɛ*_*r*_ = 4.4), and design frequency of 2 GHz. The authors stated that FR-4 substrate has a better effect on the miniaturization of coupler compared to Roger RT due to its higher dielectric constant. However, miniaturization is not a concern in the design at high frequency. The components^7,14,15^ designed on FR-4 substrate due to its low cost, with FR-4 having a high loss tangent. Since FR-4 displays a high loss tangent, it may degrade the Q-factor of the design, which leads to the degradation of performance compared to the performance of the designs with RO4003C^6^ and RT5870^16^. Authors have added that the measured performance is also degraded due to several other factors, such as conduction losses and dielectric losses^7, 17^. Hence, FR-4 is not suitable for high-frequency design due to its high dissipation factor, which results in higher losses as the frequency increases^18^. Therefore, the FR-4 substrate is not considered suitable for high operating frequency design. Thus, most of the researchers are having a tendency to select the high-performance substrate material over FR-4 substrate for high frequency design due to this impact of high loss tangent, which will significantly affect the performance of the circuit. The dielectric losses of the substrates are closely related to the dissipation factor and the loss tangent, which are proportional to the frequency^17^. Despite, loss tangent seems to be a clear factor in the substrate selection, other dielectric properties’ effects toward the design such as dielectric constant and Q-factor are required to be carefully studied at high frequency together with loss tangent to ensure a good performance can be accomplished particularly for microstrip implementation. The effects due to dielectric constant and Q-factor cannot be known from the manufacturer datasheet without systematic study and analysis.

The remainder of this paper is organized in the following manner. Next section introduces the analysis of different substrates having different properties with respect to the dielectric constant, loss tangent, and Q-factor associated with dielectric properties and their relationship to BLC performance was observed. The enhanced BLC design was then proposed with the implementation of microstrip-slot stub impedance at each port’s transmission line. The performance was simulated and measured. It was then compared to the conventional design and other designs using different techniques, and discussed thoroughly.

## Methods

Selecting the best substrate to be incorporated into the design is crucial, especially at higher operating frequencies used in 5G wireless communication applications. Therefore, in this study, the analysis of a single-section planar BLC with different substrates was conducted. Questions arise regarding which substrate among the available high-performance substrates offers the best performance for the 3-dB BLC design at the designated frequency. The analysis in this study is based on the dielectric constant, loss tangent, Q-factor and their relationship to BLC performance.

### Analysis of different substrates

The characteristics of different substrates can affect the overall performance of the design. Four different substrates were selected for analysis, including RT5800, RO4350, TMM4 and RT6010, which were chosen due to their excellent performance at higher frequencies. Each of the four substrates has a different dielectric constant and loss tangent, while the thickness of the substrate was fixed at 0.254 mm. The properties of each substrate are summarized in Table 1.

**Table 1:** The substrates with the different materials and dielectric properties.

Substrate	Material	Dielectric Constant, *ɛ*_*r*_	Loss Tangent, tan δ	Thickness *h* (mm)	Thermal Coefficient (ppm/°C)	Condition (GHz)
RT5880	Glass microfiber reinforced PTFE composite	2.2	0.0009	0.254	− 125	8–40
R04350	Woven glass reinforced hydrocarbon ceramic	3.66	0.0037	0.254	+ 50	8–40
TMM4	Ceramic thermoset polymer composite	4.7	0.0020	0.254	+ 15	8–40
RT6010	Ceramic PTFE composite	10.7	0.0023	0.254	− 425	8–40

RT5880, which is made of glass microfiber reinforced polytetrafluoroethylene (PTFE) composite, displayed the lowest dielectric constant (2.2) among all the chosen high laminating frequency substrates, the lowest loss tangent (0.0009), and a negative thermal coefficient of − 125 ppm/°C^19^. Since RT5880 has a low dielectric constant, it is suitable for high frequency applications because the electrical losses and dispersion is considered to be minimal^19^. Meanwhile, the RO4350 substrate, which is a woven glass reinforced hydrocarbon ceramic laminate displayed second-lowest dielectric constant (3.66), the highest loss tangent (0.0037), and the highest positive thermal coefficient of + 50 ppm/°C^20^. This substrate provided tight control of the dielectric constant and displayed low loss^19^. The third substrate was TMM4, which is composed of a ceramic thermoset polymer composite with a dielectric constant (4.7), the second-lowest loss tangent (0.0020), and a low thermal coefficient of + 15 ppm/°C^21^. The electrical and mechanical properties of TMM4 laminates combine many of the benefits of both ceramic and traditional (PTFE) microwave circuit laminates^21^. Finally, the RT6010 substrate had the highest dielectric constant (10.7) with negative thermal coefficient of − 425 ppm/°C^18^. Furthermore, this substrate also displayed low loss with loss tangent of 0.0023^22^.

Generally, when selecting a dielectric material during the design process, two parameters are considered, including the dielectric constant and loss tangent. The loss tangent, tan *δ* defines the measure of signal loss as the signal propagates through the transmission line, and can be expressed as (1)^23,24^:

1 $$\tan \delta = \frac{{\omega \varepsilon_{r}^{\prime \prime } + \sigma }}{{\omega \varepsilon_{r}^{\prime } }}$$

where $\varepsilon_{r}^{\prime }$ and $\varepsilon_{r}^{\prime \prime }$ are the real and imaginary part of the complex relative permittivity, $\varepsilon_{r}^{*}$, respectively. Meanwhile, *ω* and *σ* are angular frequency and conductivity, respectively with conditions of $\varepsilon_{r}^{{\prime \prime }} \ge 0$ and $\varepsilon_{r}^{\prime } \gg \varepsilon_{r}^{\prime \prime }$. The real part of $\varepsilon_{r}^{*}$, which is $\varepsilon_{r}^{{\prime }}$ associated to the ability of a material to store the incident electromagnetic (EM) energy through wave propagation, while, the imaginary part of $\varepsilon_{r}^{*}$ is denoted by $\varepsilon_{r}^{{\prime \prime }}$ related to the degree of EM energy losses in the material. Thus, $\varepsilon_{r}^{\prime }$ and $\varepsilon_{r}^{\prime \prime }$ are also known as the dielectric constant, *ɛ*_*r*_ and the loss factor, respectively. At high frequencies that were considered in this proposed work, the substrate’s loss tangent, tan *δ* can be simplified to $\varepsilon_{r}^{\prime \prime } /\varepsilon_{r}^{\prime }$. It is also known as the dissipation factor that describes the angle difference between capacitance current and voltage. Hence, a lower loss tangent is required in the substrate to ensure low dielectric loss and low dielectric absorption^25^. These two parameters, *ɛ*_*r*_ and tan *δ* are directly related to the Q-factor due to the dielectric, *Q*_*d*_ that can be expressed as (2)^26^:

2 $$Q_{d} = 27.3\frac{{\sqrt {\varepsilon_{eff} } }}{{\alpha_{d} \lambda_{0} }}$$

where the $\varepsilon_{eff}$*,* λ_0_ and *α*_*d*_ are the effective dielectric constant, the wavelength in the air and dielectric loss, respectively. The *ε*_*eff*_ can be defined by (3)^23^:

3 $$\varepsilon_{eff} = \frac{{\varepsilon_{r} + 1}}{2} + \frac{{\varepsilon_{r} - 2}}{2}\frac{1}{{\sqrt {1 + 12h/W_{m} } }}$$

where *h* and *W*_*m*_ are the thickness of the substrate and the width of the microstrip transmission line, respectively, while, the dielectric loss, $\alpha_{d}$ can be expressed as (4)^26^:

4 $$\alpha_{d} = 27.3\frac{{\varepsilon_{r} \left( {\varepsilon_{eff} - 1} \right)\tan \delta }}{{\sqrt {\varepsilon_{eff} \left( {\varepsilon_{r} - 1} \right)\lambda_{o} } }}$$

**Figure 1:**
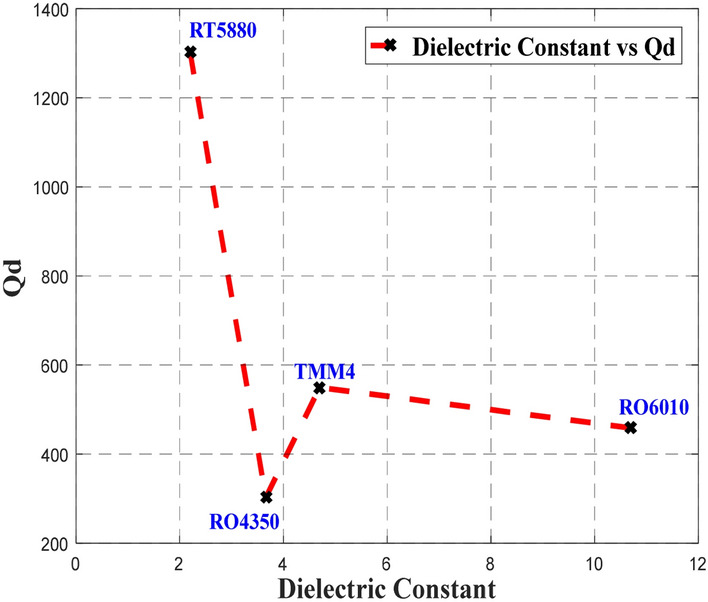
The relation between the Q-factor associated with the dielectric properties, *Q*_*d *_and dielectric constant of the different substrates.

**Figure 2:**
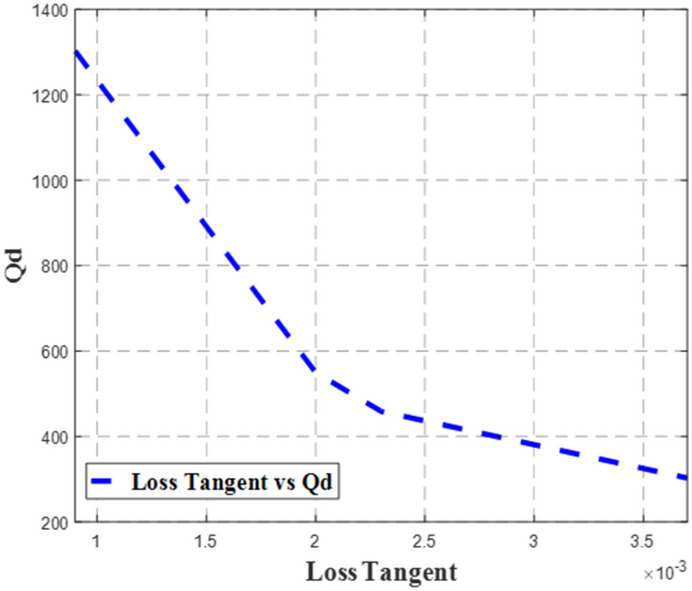
The relation between the Q-factor associated with the dielectric properties, *Q*_*d*_ and loss tangent, tan *δ*.

Thereafter, an analysis of the Q-factor associated with the material’s dielectric properties, *Q*_*d*_ was performed through calculation implementing (2)–(4) to observe the effect of different substrates, including RT5880, RO4350, TMM4 and RT6010, which have a different dielectric constant, *ɛ*_*r*_ and loss tangent, tan *δ*. The width of the microstrip transmission line, *W*_*m*_ that correspond to 50Ω was used in this analysis. The relationship between *Q*_*d*_ and the different *ɛ*_*r*_ of RT5880, RO4350, TMM4 and RT6010 substrates, and the relationship between *Q*_*d*_ and tan *δ* are depicted in Figs. 1 and 2.

As presented in Fig. 1, RT5880, RO4350, TMM4 and RT6010 substrates have *Q*_*d*_ values of 1302.79, 302.56, 549.70 and 458.69, respectively. It is important to note that *Q*_*d*_ generally decreases as the value of $\varepsilon_{r}^{{}}$ increases as depicted by the proportional correlation between *Q*_*d*_ and $\varepsilon_{eff}$ , which is calculated using Eq. (2), where RT5880 has the highest *Q*_*d*_ and the lowest $\varepsilon_{r}^{{}}$. However, this trend does not apply to RO4350 that had the second-lowest $\varepsilon_{r}^{{}}$ and the lowest performance of *Q*_*d*_. Hence, by referring back to Eqs. (2) and (4), setting aside the dielectric constant, $\varepsilon_{r}^{{}}$, *Q*_*d*_ is inversely proportional to dielectric loss, *α*_*d*_. Consequently, this *α*_*d*_ is determined by $\varepsilon_{r}^{{}}$ and tan *δ* as expressed in Eq. (4). Since *α*_*d*_ is proportional to tan *δ*, and *Q*_*d*_ is inversely proportional to *α*_*d*_, thus *Q*_*d*_ is inversely proportional to tan *δ*. Meanwhile since *α*_*d*_ is a function of $\varepsilon_{r}^{{}}$, the influence of $\tan \delta$ towards *Q*_*d*_ is greater compared to $\varepsilon_{r}^{{}}$, which can be observed from the plot in Fig. 2. Referring to Fig. 2, the value of *Q*_*d*_ decreases as the value of tan *δ* increases. Therefore, even though RO4350 has the second-lowest $\varepsilon_{r}^{{}}$, it has the highest $\tan \delta$ among the substrates, which led to the lowest *Q*_*d*_ performance.

Generally, this behavior can be explained by the characteristics of the $\varepsilon_{r}^{{}}$ that is influenced by ionic or electronic polarization, which generates *α*_*d*_ in the presence of electromagnetic wave^27^. The increasing value of $\varepsilon_{r}^{{}}$ provides a higher *α*_*d*_ value as the electric field intensity inside the dielectric layer increases^23^. RT5880 and RT6010 have polytetrafluoroethylene (PTFE) in their composition, while TMM4 has a polymer with low thermal conductivity, an excellent coefficient of thermal expansion (CTE), and low processing temperature, which results in low *α*_*d*_^28^ as presented in Table 1. While, glass is a good thermal and homogeneity insulator, it also displays a high dielectric loss^28^. To obtain a lower *α*_*d*_ value and maintain the advantages of glass, glass-reinforced ceramics can be used as displayed by R04350^28^. In any event, the dielectric loss of R04350 is still higher than RT5880, TMM4 and RT6010. Following this analysis of the four substrates, further analysis was performed by employing a single-section planar 3-dB BLC design.

### Analysis of BLC using different substrates

**Figure 3:**
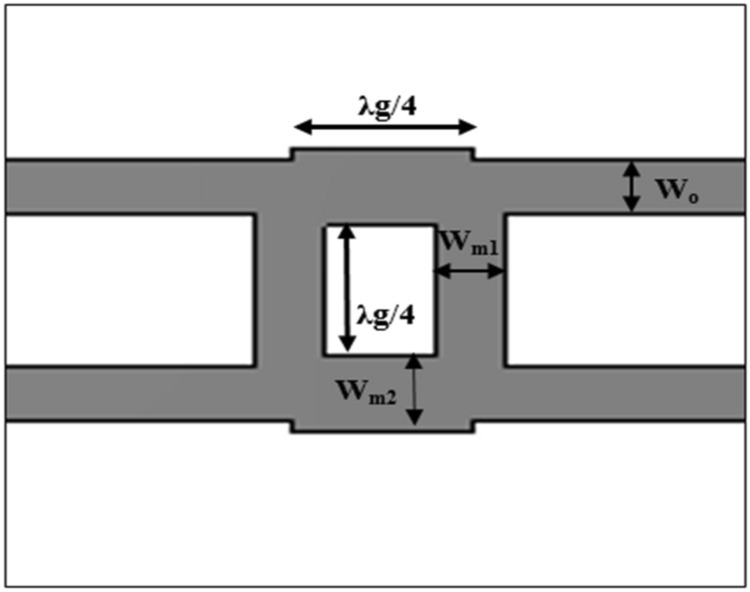
The design of the single-section 3-dB branch line coupler.

Figure 3 presents the design of the single-section 3-dB BLC. The common microstrip equation is denoted as (5), which was used to compute *W*_*o*_, *W*_*m*1_ and *W*_*m*2_, where *W*_*o*_ and *W*_*m*2_ refer to the characteristics of 50Ω, while *W*_*m*1_ refer to those of 35Ω^23^:

5 $$\frac{{W_{m} }}{h} = \frac{2}{\pi }\left[ {B - 1 - \ln \left( {2B - 1} \right) + \frac{{\varepsilon_{r} }}{{2\varepsilon_{r} }}\left\{ {\ln \left( {B - 1} \right) + 0.39 - \frac{0.61}{{\varepsilon_{r} }}} \right\}} \right]$$

where constant *B* can be expressed as (6)^23^:

6 $$B = \frac{377\pi }{{2Z_{0} \sqrt {\varepsilon_{r} } }}$$

where *Z*_0_ is the characteristic impedance.

The guide wavelength, λ_g_ was then determined by (7)^29^:

7 $$\lambda_{g} = \frac{c}{{f\sqrt {\varepsilon_{eff} } }}$$

where *c* and *f* are the speed of light and design frequency, respectively. The properties for each substrate were used to design the 3-dB BLC, and the dimensions of the designed couplers were computed and optimized, which are summarized in Table 2. The performance of each BLC was then assessed based on S-parameters, phase difference and bandwidth.

**Table 2:** Dimensions of single-section 3-dB BLCs using different substrates.

Parameters	Substrates/dimensions (mm)			
	RT5880	RO4350	TMM4	RT6010
*W*_*m*1_	0.8	0.65	0.5	0.25
*W*_*m*2_	1.1	0.9	0.6	0.5
*W*_*o*_	0.8	0.65	0.5	0.25
λ_*g*_/4	2.12	1.71	1.54	1.08

**Figure 4:**
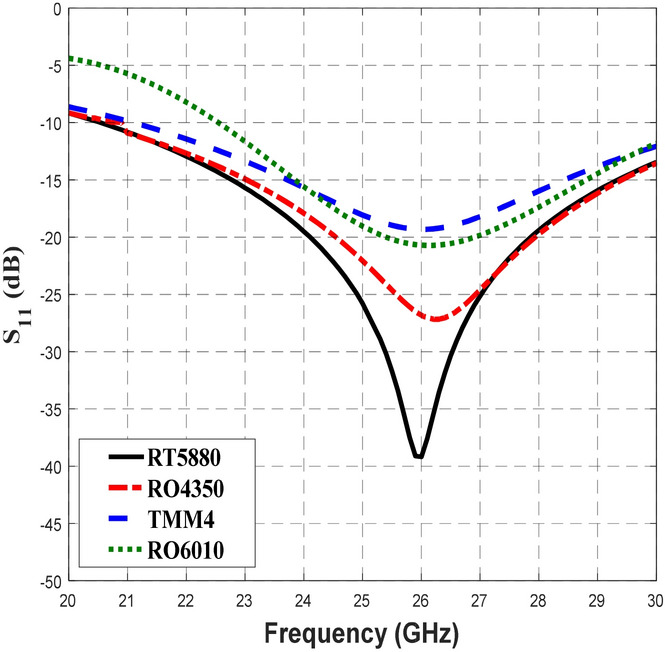
The reflection coefficients, S_11_ of the 3-dB BLC with different substrates.

The performance of each BLC was then assessed based on S-parameters, phase difference and bandwidth via simulation through the use of Computer Simulation Technology (CST) Microwave Studio software. Transient Solver tool was utilized with frequency range setting between 20 to 30 GHz and open boundary condition to calculate the energy transmission between various ports of the design structure. Figure 4 illustrates the reflection coefficient performance, S_11_ of the designed BLC with different substrates, which revealed that the S_11_ of the BLC designed with the RT5880 substrate was less than − 10 dB within a frequency range of 20.54–30 GHz. Meanwhile, the BLC design that employed the RO4350 substrate showed the performance of S_11_ was below − 10 dB across 21–30 GHz. In addition, the use of TMM4 and RT6010 offered S_11_ values that were less than − 10 dB in the ranges of 21.1–30 GHz and 22.55–30 GHz, respectively. Hence, the best S_11_ performance with the relatively broadest bandwidth and lowest S_11_ at 26 GHz, which is shown by the design that employed RT5880, which has the lowest $\varepsilon_{r}^{{}}$ and lowest tan *δ* among all four substrates was expected to have the lowest loss.

**Figure 5:**
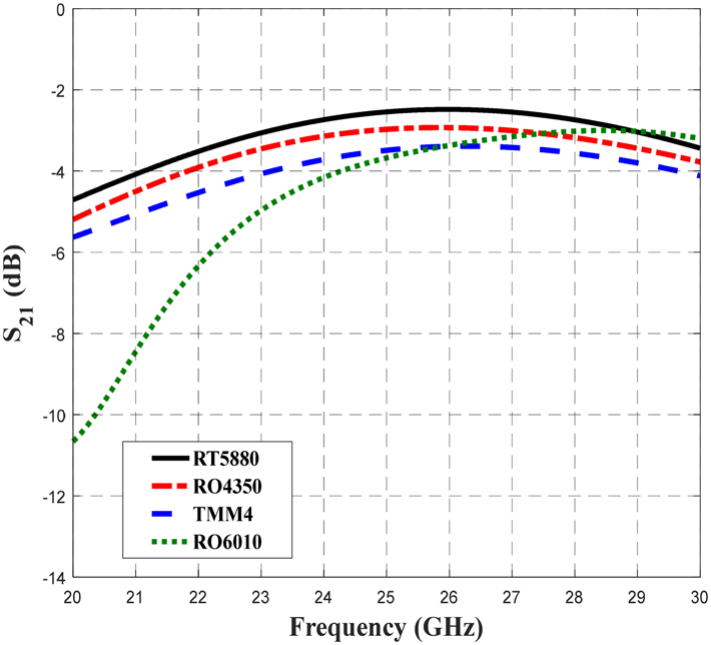
The transmission coefficients, S_21_ of the 3-dB BLC design using different substrates.

**Figure 6:**
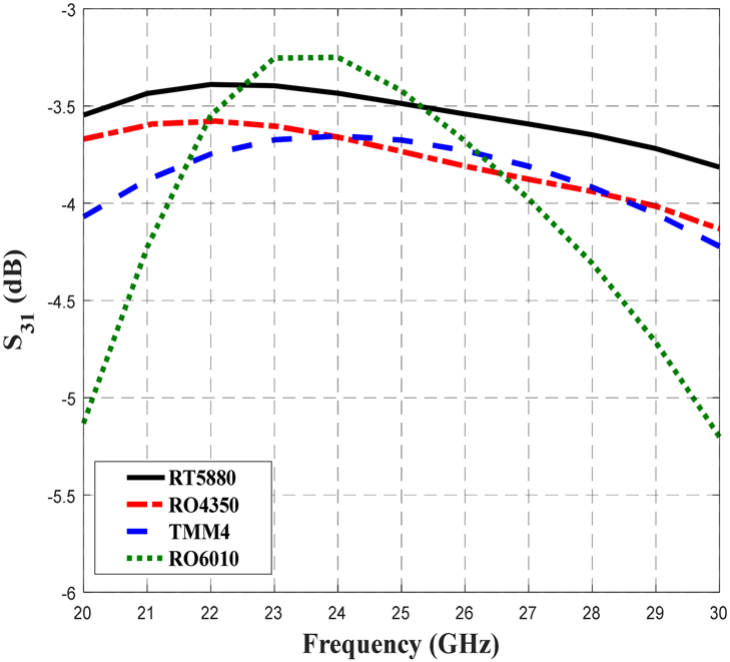
The coupling performance, S_31_ of the BLC design by using different substrates.

**Figure 7:**
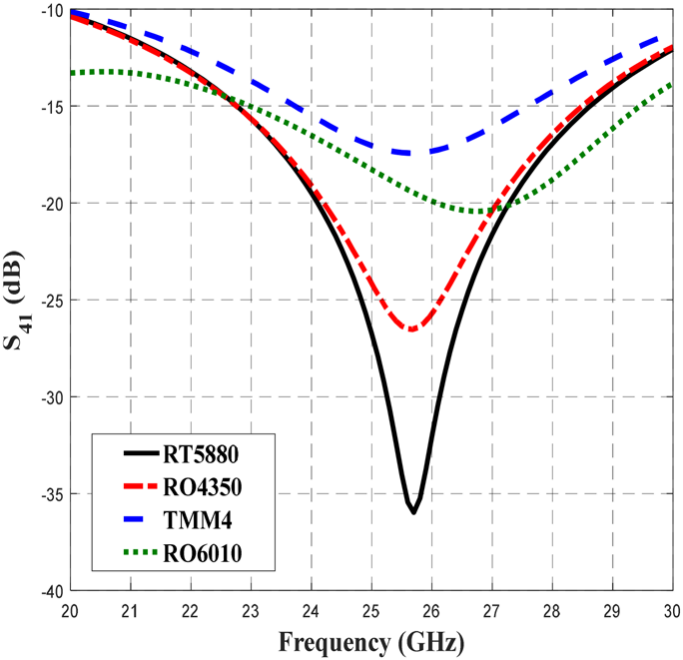
The S_41_ performance of the 3-dB BLC design with different substrates.

Figure 5 shows the transmission coefficient of S_21_ when different substrates were used in the design of the BLC. Similar S_21_ performance of − 3 dB with ± 1 dB deviation were obtained for RT5880, RO4350, TMM4, and RT6010 for slightly different frequency ranges of 21.14–30 GHz, 21.9–30 GHz, 23.18–30 GHz and 24.28–30 GHz, respectively. Compared to S_11_ performance, BLC design with RT5880 displayed the widest frequency range of 8.86 GHz with S_21_ performance of − 3 dB ± 1 dB. Meanwhile Fig. 6 depicts the coupling output, S_31_ that specifies the ratio of input power, P_1_ to the coupled power, P_3_ for BLC design that utilized different substrates. The results of our analysis indicated that the performance of S_31_ was –3 dB ± 0.9 dB within a frequency range of 20–30 GHz when RT5880 substrate was used in the design, while, the coupling performance was –3 dB ± 1 dB when the RO4350 substrate was used in a range of 20–28.74 GHz. Furthermore, similar performances of S_31_ were achieved when the design utilized TMM4 and RT6010 substrates, which were − 3 dB ± 1 dB in a frequency range of 20.34–28.62 GHz and 21.28–27.07 GHz, respectively. Hence, a coupling coefficient of 3-dB with the lowest deviation across the widest frequency range was achieved by the BLC design utilized onto RT5880 substrate. The next important analysis is associated with S_41_ performance, which involves the responses obtained from the BLC design with different substrates as depicted in Fig. 7. In this design, the lowest isolation performance was set to be 10 dB. As shown in Fig. 7, the performance of S_41_ was less than − 10 dB within a frequency range of 20–30 GHz for the design that employed all substrates. In this analysis, the lowest S_41_ performance at 26 GHz shown by the design that employed RT5880.

**Figure 8:**
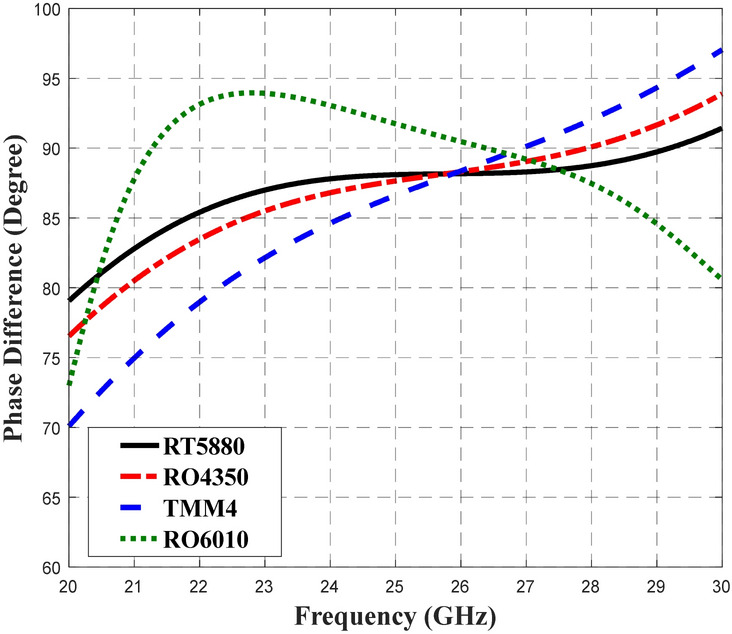
The phase difference performance of the 3-dB BLC design with the use of different substrates.

Therefore, the analysis proceeded to consider the phase difference between output ports. In this design, the deviation of the phase difference between the output ports was set to ± 2° from the ideal of 90°. Based on the phase difference analysis shown in Fig. 8, a BLC phase difference of 90° ± 2° are demonstrated by designs that employed RT5880, RO4350, TMM4 and RT6010 substrates across slightly different frequency ranges of 24.52–30 GHz, 25.52–29.17 GHz, 25.5–28 GHz and 24.81–27.73 GHz, accordingly. Similarly, as in the analysis of S_11_, S_21_, S_31_ and S_41,_ the design with RT5880 displayed the best phase performance across the widest frequency range, which is likely because it has the lowest $\varepsilon_{r}^{{}}$ and lowest tan *δ*. The performances of S_11_, S_21_, S_31_, S_41_ and the phase difference between output ports are summarized in Table 3. The Q-factor associated with the material’s dielectric properties, *Q*_*d*_ that was obtained through the analysis of those dielectric properties is presented in Table 3 for further comparison and analysis.

**Table 3:** The performance of 3-dB BLC design with different substrates and the respective *Q*_*d*_*.*

Parameters	Substrates/dimensions (mm)			
	RT5880	RO4350	TMM4	RT6010
S_11_ (dB)	< − 10	< − 10	< − 10	< − 10
S_21_ (dB)	− 3 ± 1	− 3 ± 1	− 3 ± 1	− 3 ± 1
S_31_ (dB)	− 3 ± 0.9	− 3 ± 1	− 3 ± 1	− 3 ± 1
S_41_ (dB)	< − 10	< − 10	< − 10	< − 10
Phase Difference (Degree)	90 ± 2	90 ± 2	90 ± 2	90 ± 2
Operating Frequency (GHz)	24.52–30	25.52–28.74	25.5–28	24.81–27.07
Bandwidth, GHz (%)	5.48 (21.1)	3.22 (12.4)	2.5 (9.6)	2.26 (8.7)
*Q*_*d*_	1302.79	302.56	549.70	458.69

Table 3 shows that the widest bandwidth performance of 5.48 GHz (21.1%) was achieved when the RT5880 substrate was used in the BLC design. Referring to Table I, RT5880 has the lowest $\varepsilon_{r}^{{}}$ of 2.2 and lowest $\tan \delta$ of 0.0009 which resulted in the highest* Q*_*d*_ of 1302.79. Meanwhile, RT6010 displayed the narrowest bandwidth performance of 2.26 GHz (8.7%) and it has the highest $\varepsilon_{r}^{{}}$ of 10.7 and second-highest $\tan \delta$ of 0.0023, which lead to a lower *Q*_*d*_ of 458.69. The RO4350 substrate with the highest $\tan \delta$ of 0.0037 and the second-lowest $\varepsilon_{r}^{{}}$ of 3.6 and displayed the lowest *Q*_*d*_ of 302.56, though it also had the second-widest bandwidth performance of 3.22 GHz (12.4%). Even though a lower $\tan \delta$ significantly contributes to a higher *Q*_*d*_ compared to $\varepsilon_{r}^{{}}$, results indicated that the $\varepsilon_{r}^{{}}$ is a primary factor in the determination of optimal bandwidth performance with an inversely proportional relationship. The $\varepsilon_{r}^{{}}$ of a material represents the ability of that material to store electrical energy in the presence of an electrical field, whereas, when the frequency increases, the losses in the substrate begins to reduce the ability of the dielectric material to store energy. Therefore, it can be concluded that the bandwidth performance increases as the dielectric constant decreases, while the high dielectric constant substrate may lose its ability of storing energy. Thus, based on the results of our analysis, the substrate with a low dielectric constant and a low $\tan \delta$, which contribute to the respective high bandwidth and high Q-factor is the most suitable for 5G applications at high frequencies, and in this case, a design frequency of 26 GHz that uses the RT5880 substrate was selected.

### Design of 3-dB BLC with microstrip-slot stub

**Figure 9:**
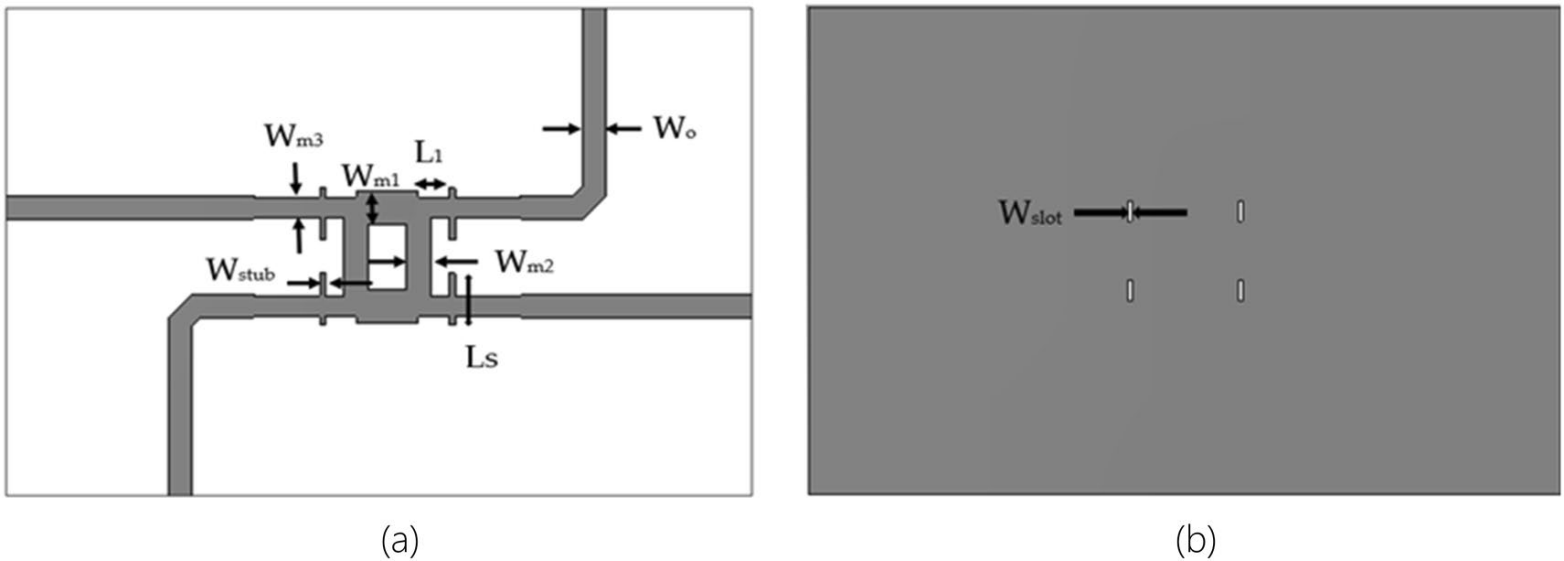
The proposed 3-dB BLC with the implementation of microstrip-slot stub at each port; (**a**) top view and (**b**) bottom view.

This section discusses the proposed wideband 3-dB BLC design, as depicted in Fig. 9, with the implementation of a microstrip-slot stub for bandwidth improvement over that of conventional BLC designs, as shown in Fig. 3 by using CST Microwave Studio with the utilization of Transient Solver tool, frequency range setting between 20 to 30 GHz and open boundary condition. The best substrate was RT5880 based on the analysis of its dielectric properties, and was thus chosen for the design. The proposed microstrip-slot stub impedance was placed at each port at a distance, *L*_1_ from the BLC. By tuning these microstrip-slot stub impedances, better matching can be achieved to ensure maximum power is transferred from the source, and a minimum signal is reflected from the load, which consequently enhances the bandwidth^30,34^.

Generally, the input impedance of the stub, *Y*_*in*_ can be written as (8)^31^:

8 $$Y_{in} = jY_{0} \tan \theta_{stub}$$

where *Y*_*0*_ and $\theta_{stub}$ are the stub admittance and the electrical length of the stub, respectively, and the $\theta_{stub}$ can be expressed as (9)^31^:

9 $$\theta_{stub} = \beta L_{s} = \frac{{\omega_{0} }}{{V_{pstub} }}L_{s}$$

where *ω*_0*,*_* L*_*s*_ and *V*_*pstub*_ are the angular frequency, the length of stub and the phase velocity of the stub, respectively. By comparing *Y* = *ωC* to Eq. (8), the length of the stub, *L*_*s*_ can be obtained in (10)^31^:

10 $$\omega_{0} C = \frac{1}{{Z_{stub} }}\tan \left( {\frac{{\omega_{0} }}{{V_{pstub} }}} \right)L_{s}$$

where *Z*_*stub*_ is the characteristic impedance of the stub. It was stated that junction discontinuities can be avoided when the length of stub impedance is half the wavelength^28^. However, the parameters still need to be optimized to achieve optimal performance. To achieve optimal performance, a stub with a higher impedance is required^32^.

Furthermore, stub impedance can form reflection zeroes at equal distances on both sides of the ports^30^. The distance of the stub impedance of the proposed BLC design is defined as *L*_1._ Referring back to the common matching technique that employs the stub^23^, the load impedance, *Z*_*L*_ representing the BLC can be expressed as (11):

11 $$Z_{L} = \frac{1}{{Y_{L} }} = R_{L} + jX_{L}$$

where *Y*_*L*_, *R*_*L*_ and *X*_*L*_ are the load admittance, the real part of load impedance and the imaginary part of load impedance, respectively. Therefore, the impedance at a distance, *L*_1_ from the load (BLC) is given in the (12) and (13):

12 $$Z_{L1} = Z_{0} \frac{{\left( {R_{L} + jX_{L} } \right) + jZ_{0} t}}{{Z_{0} + j\left( {R_{L} + jX_{L} } \right)t}}$$

and

13 $$t = \tan \beta L_{1}$$

Let the admittance of stub impedance at a distance, *L*_1_ be expressed as (14):

14 $$Y_{L1} = G + jB = \frac{1}{{Z_{L1} }}$$

where parameters *G* and *B* can be defined by (15) and (16), respectively, by using (13) and (14):

15 $$G = \frac{{R_{L} \left( {1 + \tan^{2} \beta L_{1} } \right)}}{{R_{L}^{2} \left( {X_{L} + Z_{0} \tan \beta L_{1} } \right)^{2} }}$$

and

16 $$B = \frac{{R_{L}^{2} \tan \beta L_{1} - (Z_{0} - X_{L} \tan \beta L_{1} )\left( {X_{L} + Z_{0} \tan \beta L_{1} } \right)}}{{Z_{0} \left[ {R_{L}^{2} \left( {X_{L} + Z_{0} \tan \beta d} \right)^{2} } \right]}}$$

Then, by equating $G = Y_{0} = 1/Z_{0}$ and from (13)^23^,

17 $$\frac{1}{{Z_{0} }} = \frac{{R_{L} \left( {1 + t^{2} } \right)}}{{R_{L}^{2} + \left( {X_{L} + } \right)}}$$

Therefore, the value of *t* can be expressed as (18):

18 $$t = \left\{ {\begin{array}{*{20}l} {\frac{{X_{L} \pm \sqrt {\frac{{R_{L} \left[ {\left( {Z_{0} - R_{L} } \right)^{2} + X_{L}^{2} } \right]}}{{Z_{0} }}} }}{{R_{L} - Z_{0} }},} \hfill & {for\quad R_{L} \ne Z_{0} } \hfill \\ {\frac{{ - X_{L} }}{{2Z_{0} }},} \hfill & {for\quad R_{L} = Z_{0} } \hfill \\ \end{array} } \right.$$

Thereafter, by assuming *R*_*L*_ = *Z*_*0*_ and by using $t = \tan \beta L_{1} = \tan \frac{2\pi }{\lambda }L_{1}$, the distance of stub impedance from BLC, *L*_1_ can be determined using (19):

19 $$\frac{{L_{1} }}{\lambda } = \left\{ {\begin{array}{*{20}l} {\frac{1}{2\pi }\tan^{ - 1} t,} \hfill & {for\quad t \ge 0} \hfill \\ {\frac{1}{2\pi }\left( {\pi + \tan^{ - 1} t} \right), } \hfill & {for\quad t > 0} \hfill \\ \end{array} } \right.$$

A narrow slot line is then employed at the ground plane underneath the microstrip stub to form microstrip-slot stub impedance because the use of the slot line can improve the bandwidth performance due to its slow-wave characteristic. The implementation of slot-line on the ground plane disturbs current distribution and this disturbance changes the characteristics of the transmission line, such as capacitance and inductance, to produce the slow-wave characteristics, which can increase the phase velocity delay. The characteristic impedance of the microstrip-slot stub can be determined through the use of the microstrip-slot line impedance, $Z_{m - s}$ equation as expressed in (20)^33^:

20 $$Z_{m - s} = 18.22\left( {W_{slot} } \right)^{2} + Z_{m}$$

**Figure 10:**
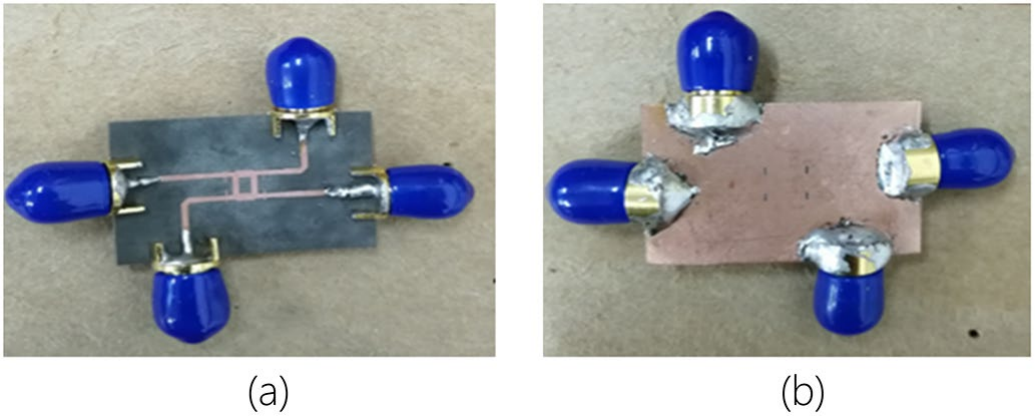
The photography of the proposed 3-dB BLC prototype with the implementation of microstrip-slot stub impedance; (**a**) front view and (**b**) back view.

Obtaining the initial dimensions through calculation, this proposed BLC was simulated and optimized accordingly. The optimized dimensions of the coupler, as depicted in Fig. 9 were *W*_o_ = 0.8 mm, *W*_*m*1_ = 1.09 mm, *W*_*m*2_ = 0.8 mm, *W*_*m*3_ = 0.7 mm, *W*_stub_ = 0.18 mm, *W*_slot_ = 0.15 mm, *L*_1_ = 0.65 mm, *L*_*s*_ = 0.85 mm and length of each branch, λ/4 = 2.12 mm. The dimensions of the proposed BLC are summarized in Table 4. The next objective is to verify the performance of the proposed BLC. Then, the proposed design was realized by employing the Roger RO5880 substrate with dielectric constant, *ɛ*_*r*_ of 2.2, a substrate thickness, *h* of 0.254 mm, and an overall size of 29.9 mm × 19.9 mm. Figure 10 shows the fabricated prototype of the proposed BLC with slotted-stub impedance.

**Table 4:** Dimensions of proposed 3-dB Branch Line Coupler.

Parameters	Dimension (mm)
*W*_*m*1_	1.09
*W*_*m*2_	0.8
*W*_*m*3_	0.7
*W*_*slot*_	0.15
*L*_1_	0.65
*L*_*s*_	0.85
*W*_*o*_	0.8
λg/4	2.12

### Measurement of 3-dB BLC with microstrip-slot stub

**Figure 11:**
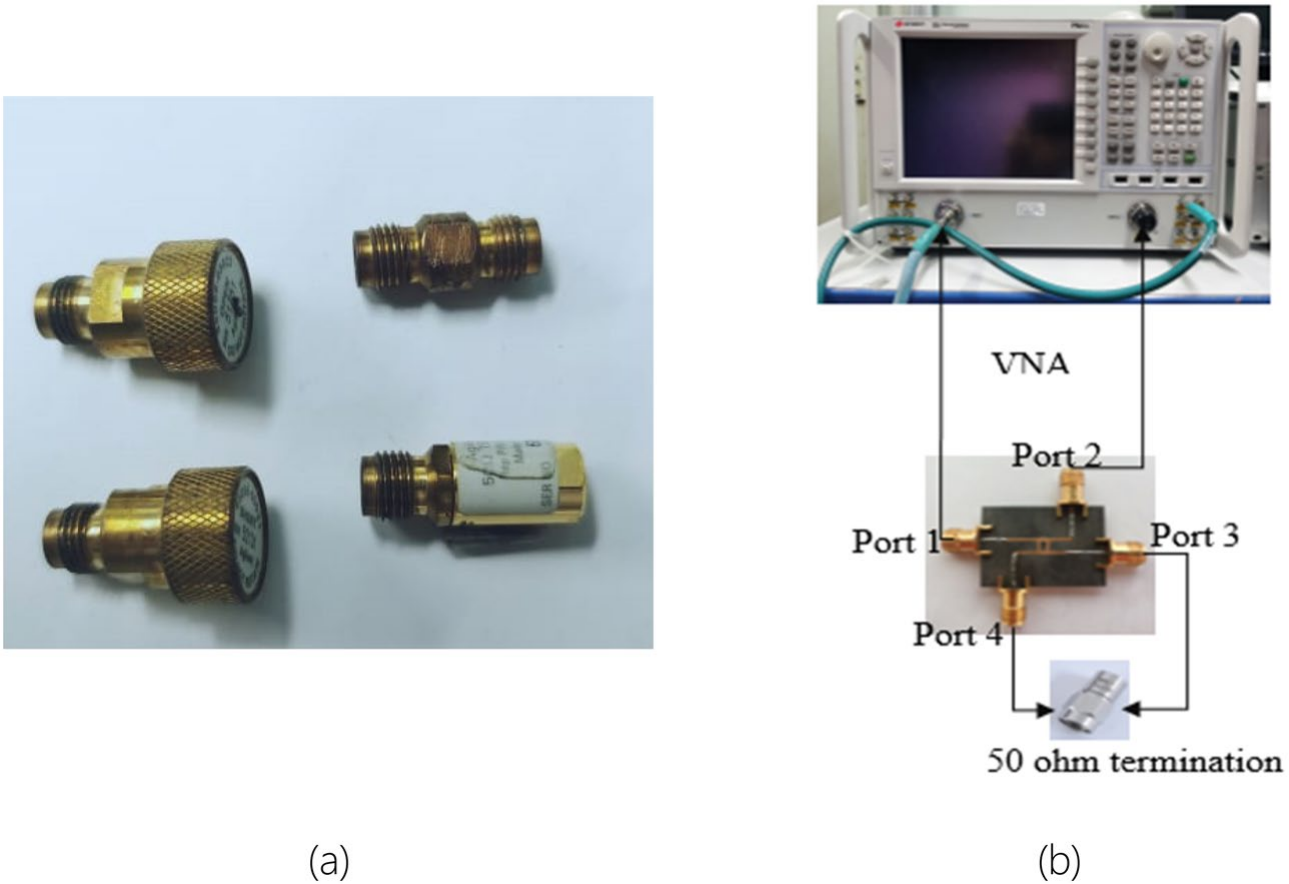
(**a**) Calibration standards^36^, and (**b**) the measurement setup of the proposed BLC using a vector network analyzer (VNA).

The measurement of the proposed 3-dB BLC with microstrip-slot stub fabricated prototype was conducted using a vector network analyzer (VNA) to verify its performance. Prior to the measurement, the two-port network calibration procedure of VNA is necessary to remove its errors. The calibration was performed using the calibration standards involving the open, short, match, and through^35^. Following the completed calibration procedure, the measurement of the proposed BLC prototype was carried out with the setup as depicted in Fig. 11. Referring to the measurement setup, the selected ports were connected directly to the VNA, while the unused ports were terminated with 50 Ω SMA termination. Thereafter, a comparison was made in terms of the simulated and measured S-parameters and phase characteristics.

## Results and discussion

**Figure 12:**
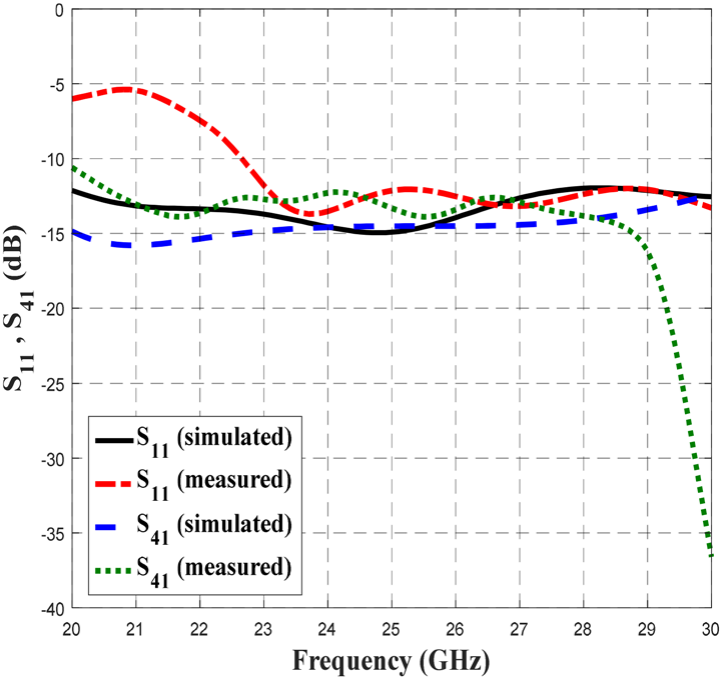
The simulated and measured S_11_ and S_41_ of the proposed BLC.

**Figure 13:**
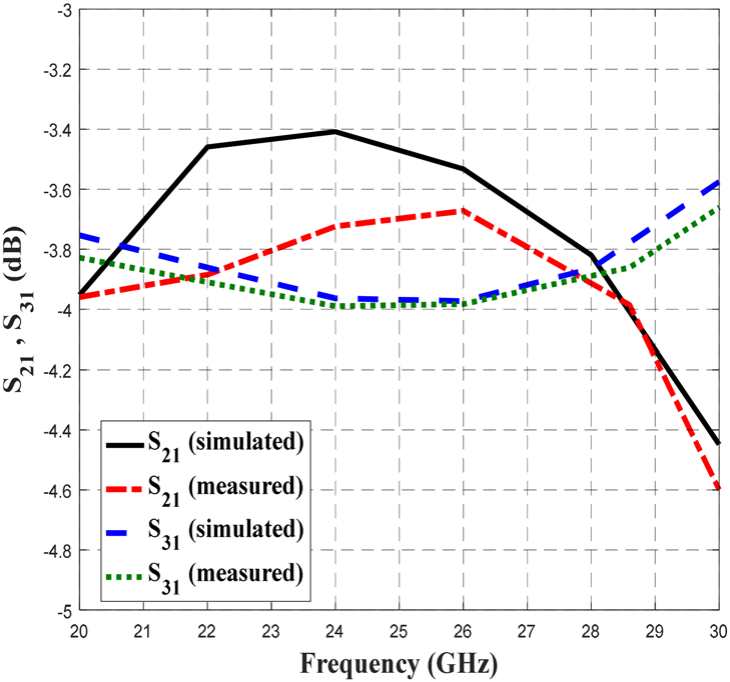
The simulated and measured S_21_ and S_31_ of the proposed BLC.

**Figure 14:**
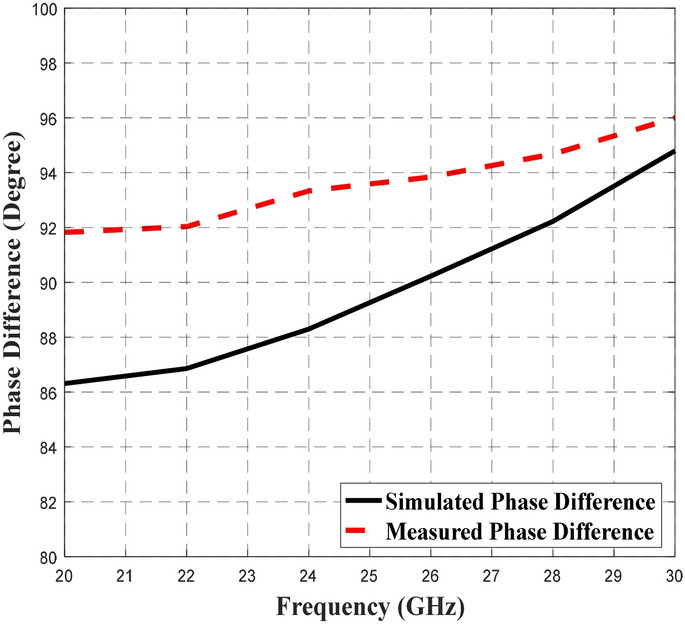
The simulated and measured phase difference of the proposed BLC.

Figures 12,13 and 14 depict the simulated and measured performance of the proposed BLC, which operated well from 20 to 28.7 GHz and 22 to 30 GHz, respectively. As shown in Fig. 12, the simulated and measured S_11_ and S_41_ values were less than − 12 dB and − 11 dB, respectively. The value of − 10 dB and below used as the specification to indicate a good transmission signal from the input port to the output port, where almost 90% of the signal is being transmitted_._ Meanwhile based on the results presented in Fig. 13, the simulated and measured transmission coefficients at the coupling port (S_31_) displayed a ± 1 dB deviation from the ideal value of 3 dB, while, the simulated and measured transmission coefficients of S_21_ depict the performance of − 3 dB ± 0.8 dB and − 3 dB ± 0.9 dB, respectively. Meanwhile, the plotted responses in Fig. 14 indicate that the simulated and measured phase differences between output ports were 90° ± 3° and 90° ± 4°, respectively. These S-parameters and phase difference performance are summarized in Table 5 to provide a clear comparison.

**Table 5:** The simulated and measured performance of the proposed 3-dB BLC design.

Parameters	Initial BLC (RT5880)	Proposed BLC	
		Simulated	Measured
S_11_ (dB)	< − 10	< − 12	< − 11
S_21_ (dB)	− 3 ± 1	− 3 ± 1	− 3 ± 1
S_31_ (dB)	− 3 ± 0.9	− 3 ± 0.8	− 3 ± 0.9
S_41_ (dB)	< − 10	< − 12	< − 11
Phase Difference	90° ± 2°	90° ± 3°	90° ± 4°
Operating Frequency (GHz)	24.52–30	20–28.7	22–30
Bandwidth (GHz)	5.48 (21.1%)	8.7 (34.8%)	8 (32%)

Based on the data in Table 5, the proposed BLC with microstrip-slot stub impedance at the ports’ transmission line appeared to result in better performance of S_11_, and S_41_ at a bandwidth was improved by approximately 2.52 GHz compared to initial single-section BLC design. Comparable transmission coefficients of S_21_ and S_31_ were observed between the proposed BLC and initial BLC designs. However, the phase difference between the output ports of the proposed BLC has deviated slightly more (± 1°) than initial BLC designs, but it was still within a reasonable performance range with respect to phase difference. Furthermore, performance of the simulated and measured BLC with microstrip-slot stub impedance were consistent with one another, along with an operating frequency that was slightly shifted. One of the main reasons that have been found affecting the measurement results was a small discrepancy in the width of the microstrip-slot stub impedance. To prove this, simulation on different widths of the microstrip-slot stub impedance was performed, analyzed, and discussed in the next sub-section.

### Parametric analysis on different widths of the microstrip-slot stub impedance

**Figure 15:**
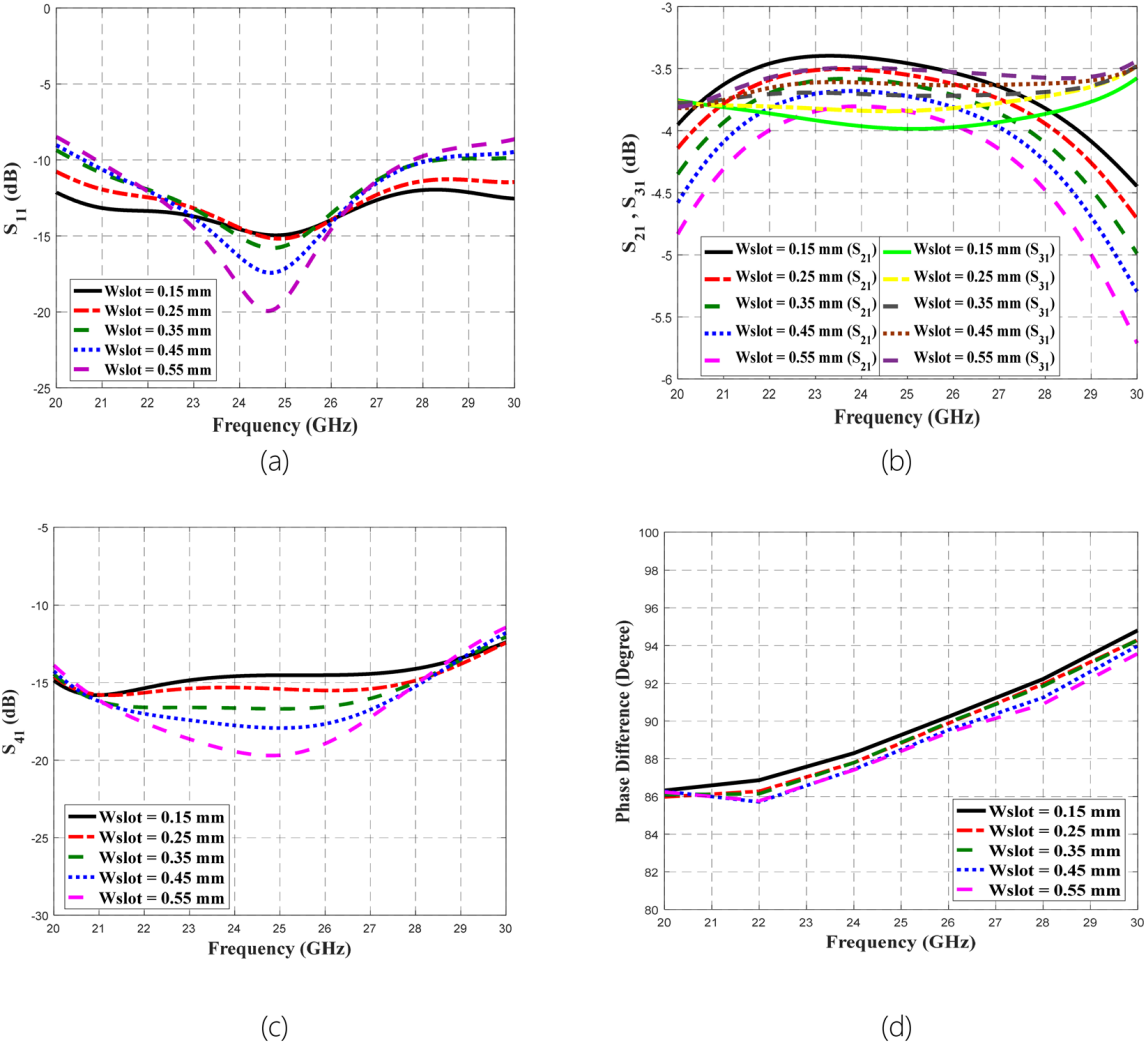
Parametric analysis on varied *W*_*slot*_ concerning (**a**) S_11_, (**b**) S_21_ and S_31_, (**c**) S_41_, and (**d**) phase difference between output ports.

Parametric analysis on different widths of the microstrip-slot stub impedance concerning its microstrip stub width, *W*_*stub*_ and slot line width, *W*_*slot*_ was performed via the use of CST Microwave Studio with a similar setting as in analysis and design in the section of Methods. Initially, the parametric analysis was started by fixing *W*_*stub*_ to its optimal dimension of 0.18 mm and varying *W*_slot_ between 0.15 mm and 0.55 mm. The effect of this varied *W*_*slot*_ was observed through S-parameters and phase difference as depicted in the following Fig. 15.

**Figure 16:**
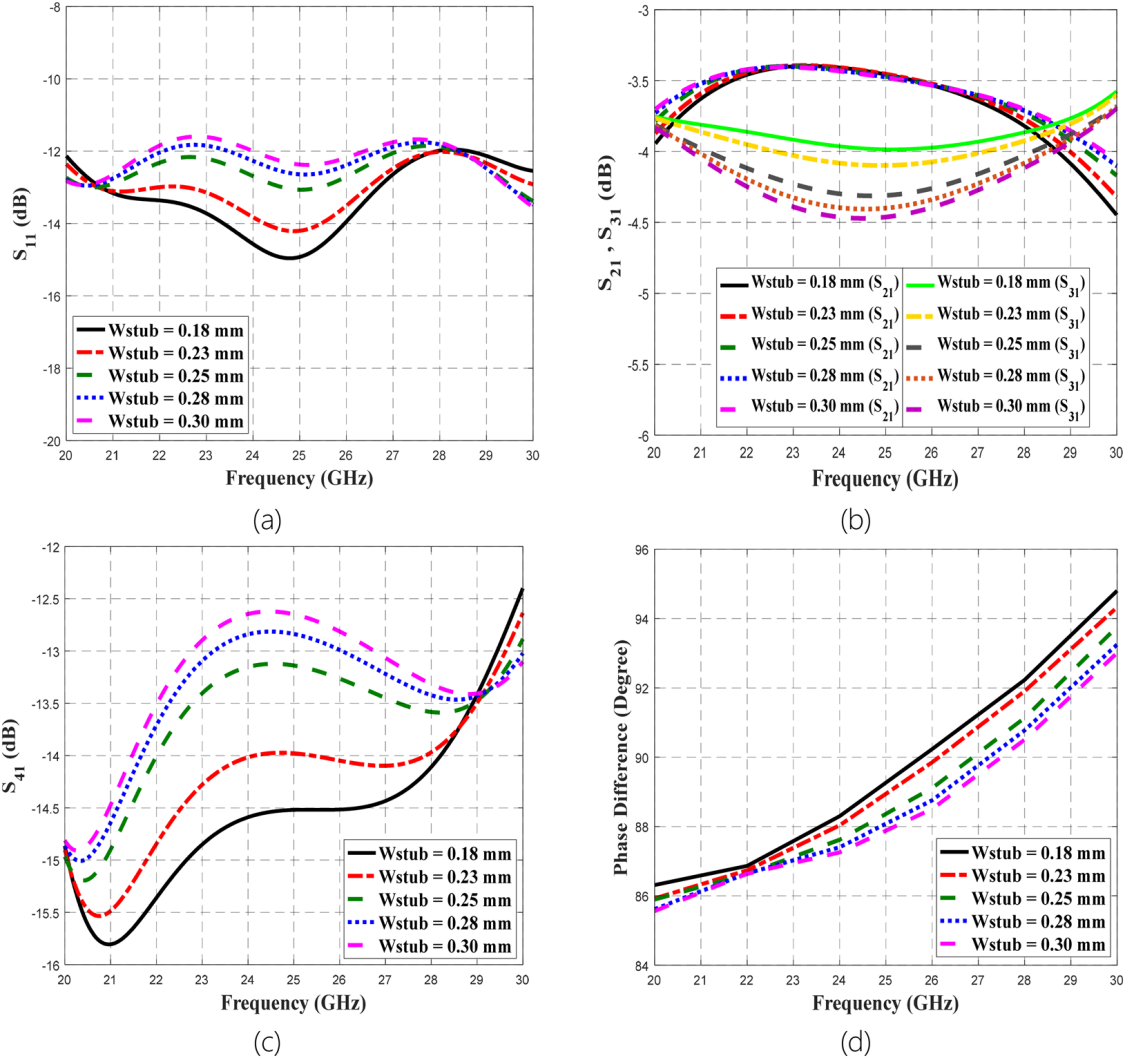
The parametric analysis on varied *W*_stub_ concerning (**a**) S_11_, (**b**) S_21_ and S_31_, (**c**) S_41_, and (**d**) phase difference between output ports.

The function of slot implementation is to broaden the bandwidth due to its slow-wave characteristics. Concerning the bandwidth performance, it shows that from the plotted graphs in Fig. 15, the broadest bandwidth was provided by the smallest value of *W*_*slot*_ (0.15 mm) compared to the largest value of *W*_*slot*_ (0.55 mm). Besides, the smallest amplitude imbalance and phase imbalance were offered by 0.15 mm *W*_*slot*_. Thus, the optimal *W*_*slot*_ dimension is 0.15 mm for this proposed coupler design. Any discrepancy from this optimal width will lead to deviation in the results of S-parameters and phase difference, in which the deviation trends can be observed through the plotted graphs. By comparing the plotted varied *W*_*slot*_ graphs to the summarized measured results in Table 5 and the assumption of fixed *W*_s*tub*_ at 0.18 mm, it can be estimated that the fabricated coupler could have 0.35 mm *W*_*slot*_ instead of 0.15 mm. Afterward, the next concern is the effect of the varied *W*_*stub*_ towards the performance of the proposed BLC by fixing *W*_*slot*_ to its optimal dimension of 0.15 mm. *W*_*stub*_ was varied from 0.18 mm to 0.30 mm in this parametric analysis, which the effects on S-parameters and phase difference are shown in Fig. 16.

The addition of stub impedance in the design is to improve the matching, which consequently enhances the bandwidth performance compared to the design without stub impedance. Hence, *W*_*stub*_ increment from 0.18 mm to 0.30 mm can be seen affecting the matching and isolation of the coupler, which noted through the plotted S_11_ and S_41_ in the respective Fig. 16 (a) and (c). Meanwhile, degradation also can be noticed for S_31_ and phase difference between output ports presented in Fig. 16 (b) and (d), correspondingly. Whilst, minimal effect due to *W*_stub_ variation can be observed for S_21_. Thus, smaller *W*_stub_ is better compared to larger *W*_stub_ with the optimal dimension of 0.18 mm. Then with the fixed 0.15 mm *W*_slot_, the plotted varied *W*_stub_ graphs were compared to the summarized measured results in Table 5. Thus, it can be predicted that the fabricated coupler could not have *W*_stub_ discrepancy from its optimal 0.18 mm. Hence from this analysis, the deviation observed from the measurement results of the proposed coupler compared to the simulation can be due to the fabricated coupler has slightly wider *W*_*slot*_ (0.35 mm) than its optimal width of 0.15 mm.

### Comparison of the proposed 3-dB BLC with microstrip-slot stub to other designs

Nonetheless, concerning its amplitude deviation, phase deviation, and operating frequency, the proposed design is compared to other coupler designs^37–39^ using different techniques. By referring to Table 6, the proposed design has comparable amplitude imbalance, phase imbalance, and bandwidth with the design based on lumped-elements and fabricated using integrated passive devise (IPD) technology on glass substrate proposed by Cayron et al.^37^. Another coupler^38^ that fabricated using IPD chip-level technology on gallium arsenide (GaAs) based substrate has higher amplitude imbalance but better phase imbalance compared to the proposed design. While two coupler designs based on the respective substrate integrated waveguide (SIW) and stripline demonstrated higher amplitude imbalance and phase imbalance with narrower bandwidth compared to the proposed design. Hence, by denoting this comparison, the good planar microstrip coupler design with a well-chosen substrate of RT5880 that has a low dielectric constant, very low $\tan \delta$, and high Q-factor as shown by this proposed design can offer very well wideband performance even though planar technology faces significant losses at high frequency.

**Table 6:** Comparison of the proposed design with other coupler designs using different techniques.

Design	Technique	Amplitude Imbalance	Phase Imbalance	Operating Frequency (GHz)
Proposed Design	Microstrip with microstrip-slot stub impedance	± 1 dB	± 4°	22–30 (BW = 8)
3 dB coupler^37^	Lumped-elements IPD fabrication (Substrate: Glass)	± 0.9 dB	± 3°	19.5–26.5 (BW = 7)
Lange coupler^38^	IPD chip-level fabrication (Substrate: GaAs)	± 1.5 dB	± 2°	26
3 dB coupler^39^	SIW	± 2.7 dB	± 6°	24–28 (BW = 4)
	Stripline	± 1.8 dB	± 6°	24–28 (BW = 4)

*BW Bandwidth.

## Conclusion

Based on the analysis of dielectric materials that lower loss tangent, tan *δ* contributes to a higher Q-factor due to dielectric properties, *Q*_*d*_, while a lower dielectric constant, *ɛ*_*r*_ results in greater bandwidth performance. Thus, to ensure a device designed at high frequency for 5G application is perform well, the substrate must be selected based on it having a low dielectric constant, *ɛ*_*r*_ , a low loss tangent, tan *δ* and a high Q-factor due to dielectric properties, *Q*_*d*_. Hence, the substrate that displayed the best performance, which was RT5880 due to its lowest *ɛ*_*r*_ of 2.2, lowest tan *δ* of 0.0009 and highest *Q*_*d*_ of 1302.79 was selected. Its use in the design was presented with a proposed wideband 3-dB BLC with microstrip-slot stub impedance and overall dimensions of 29.9 mm × 19.9 mm. The design and optimization were conducted using CST Microwave Studio, which is an electromagnetic (EM) simulator. The performances of transmission coefficients, reflection coefficients and phase characteristics of the designed coupler were obtained and analyzed. Its wideband performance at a design frequency of 26 GHz was proven via measurements of the fabricated prototype’s performance in the laboratory.
